# High-intensity interval training modifies energy supply in male artistic gymnastics and improves floor-specific but not pommel horse-specific endurance performance

**DOI:** 10.3389/fspor.2025.1601074

**Published:** 2025-10-29

**Authors:** Alexander Seemann-Sinn, Peter Rüdrich, Ingo Sandau, Falk Naundorf, Bernd Wolfarth

**Affiliations:** ^1^ Department of Sports Medicine, Humboldt University of Berlin, Berlin, Germany; ^2^ Department of Strength, Power and Technical Sports, Institute for Applied Training Science, Leipzig, Germany; ^3^ Department of Sports Medicine, Institute for Applied Training Science, Leipzig, Germany; ^4^ Department of Sports Medicine, Charité - Universitätsmedizin Berlin, Corporate Member of Freie Universität Berlin and Humboldt-Universität zu Berlin, Berlin, Germany

**Keywords:** artistic gymnastics, HIIT, gymnastic endurance, energy metabolism, VO_2_ kinetics, O_2_ deficit

## Abstract

**Introduction:**

Well-developed specific endurance performance in artistic gymnastics is a crucial foundation of successful performance. Therefore, the identification of effective training programs for gymnastics-specific endurance performance is an important factor. This study aims to analyze the effectiveness of high-intensity interval training (HIIT) on gymnastics-specific endurance performance and changes in energy supply during floor and pommel horse routines.

**Methods:**

Twenty-five male artistic gymnasts (age: 15.0 ± 1.5 years; weight: 51.2 ± 10.6 kg; height: 160.0 ± 10.4 cm) were allocated to either the experimental (n = 14) or the control group (n = 11). For 6 weeks, the intervention group completed a HIIT two times a week, integrated into the normal gymnastics training. The control group performed normal gymnastics training, including their routine programs for physical fitness development. Floor- and pommel horse-specific endurance performance was determined by the mean jump height in a repeated jump test and by the maximum number of circles in a circle test. Energy supply on the floor and pommel horse was calculated using mobile spiroergometry and the PCr-LA-O_2_ method.

**Results:**

The gain score analysis showed a significantly greater improvement in mean jump height for the intervention group, but no significant effect for the maximum number of circles. In terms of energy supply, the intervention group showed a significantly greater increase in aerobic metabolism on the floor and pommel horse. Additional correlation analyses show relationships between physiological parameters (O_2_ deficit and VO_2_ kinetics) and endurance performance as well as the modification of energy supply.

**Conclusion:**

The results of the study show that 6 weeks of semi-specific HIIT can improve floor-specific endurance performance and increase the aerobic metabolism during floor and pommel horse routines. Faster VO_2_ kinetics and the associated lower O_2_ deficit seem to be important physiological parameters for these improvements.

## 1 Introduction

Top international artistic gymnastics is characterized by an ongoing increase in the level of difficulty of the routines and, as a result, an increase in the duration and intensity of the routines (1–3). Therefore, beyond a high technical skill level, simultaneous development of well-developed gymnastics-specific muscular strength and endurance are essential to meet the increased demands of the competition (4). Regarding gymnastics-specific endurance, there are clear indications that the demands on the cardiorespiratory and metabolic system are higher and more complex than previously assumed (5). In this context, previous studies based on routine duration and blood lactate concentrations (BLC) estimated that the aerobic metabolism is utilized by 3%–8% during pommel horse, still rings, and bar routines and 20%–30% during floor routines (6, 7). Recent studies on the physiological and energetic demands on the floor, pommel horse, and still rings contradict these assumptions (8–10). According to these studies, the percentage aerobic energy supply is 23.3 ± 4.1% on pommel horse (8), 28.6 ± 4.8% on still rings (9), and 54.4 ± 6.8% on floor (10). These results are consistent with studies confirming a notable aerobic energy supply (between 43% and 49%) in other sports with similar routine durations and intensity profiles (i.e., indoor climbing, 60 s judo matches, or rhythmic gymnastics) (11–13). Due to these differences between the earlier estimates and recent findings, there is still no consensus regarding the specific energetic demands or the most effective training method for gymnastics-specific endurance performance. While some authors do not attribute any importance to aerobic metabolism (6), other authors point out that aerobic performance training should not be neglected (14–16). Currently, training regimes to improve the gymnastics-specific endurance performance focused either on strength training to improve maximal strength capabilities (17), or on high-intensity interval training (HIIT) to increase the endurance capabilities (i.e., VO_2max_, muscle endurance) (18–21). However, it is currently unclear whether HIIT improves specific endurance performance in artistic gymnastics and which physiological factors are important for achieving this kind of improvement.

**Figure 1:**
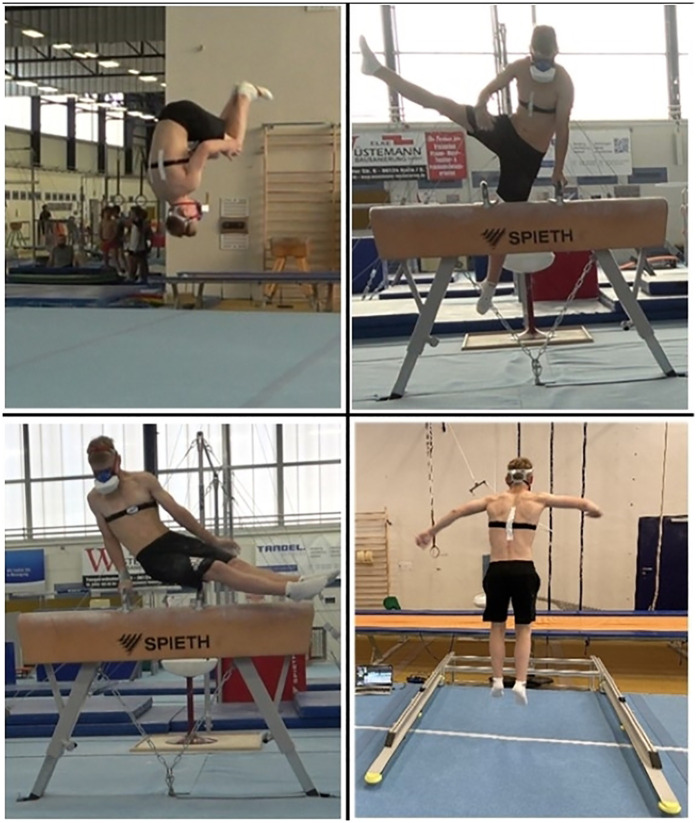
Graphical presentation of a subject performing the floor routine (top left), the pommel horse routine (top right), the maximum circle test (below left) and the repeated jump test (below right).

As mentioned previously, aerobic metabolism plays a significant role in the energy supply in artistic gymnastics. An improvement in aerobic performance can lead to a modification in energy supply and thus create an energetic power reserve at a given routine load. Increasing the proportion of aerobic metabolism could potentially save the limited high-energy creatine phosphate (PCr) and reduces lactate and H^+^ production and the breakdown of muscle glycogen (22). Reduced lactate and H^+^ production minimizes the reduction in muscle pH and thus the energetic factors of muscular fatigue caused by anaerobic lactacid metabolism, as the reduction in muscle pH interferes with biochemical and physiological processes (23). To achieve an increase in the aerobic metabolic rate, it is important to speed up the aerobic metabolism, which is determined by the oxygen uptake (VO_2_) kinetics (24). Faster VO_2_ kinetics means a smaller O_2_ deficit and therefore less PCr depletion and less production of lactate and H^+^ by the anaerobic lactic metabolism, which is associated with an increase in exercise tolerance (22, 24). The rate of VO_2_ kinetics is mainly limited by the intramuscular part (enzyme activity and content and mitochondrial volume and content) of VO_2_ (25). Notably, HIIT appears to be an effective method of speeding up VO_2_ kinetics (22, 24). Studies have shown a 17%–34% reduction in the time constant (*τ*_1_) of VO_2_ kinetics during moderate and heavy exercise for both upper and lower body exercise (22, 25–28). However, it is unclear how HIIT influences VO_2_ kinetics during gymnastic specific endurance tests and whether these effects can be transferred to achieve the desired modification of energy supply during artistic gymnastic routines.

The aim of the study was therefore to analyze the effectiveness of HIIT on gymnastics-specific endurance performance and the change in energy supply using floor and pommel horse routines as examples. Based on the theoretical positions, it was hypothesized (H1) that HIIT integrated into gymnastics training would lead to a greater improvement in performance in gymnastics-specific endurance tests compared to normal gymnastics training. The second hypothesis (H2) was that HIIT integrated into gymnastics training would lead to a greater increase in the relative aerobic energy component and a greater reduction in the anaerobic energy component of gymnastics routines on the floor and pommel horse compared to normal gymnastics training. In order to examine the possible factors of the effectiveness of HIIT, it was examined whether there are correlations between physiological factors (e.g., O_2_ deficit) and an improvement in endurance performance. In this context, it was hypothesized (H3) that the O_2_ deficit correlates with the performance parameters in the gymnastics-specific endurance tests. The final hypothesis (H4) tested was that the O_2_ deficit correlates with the aerobic and anaerobic energy contribution of the floor and pommel horse routines.

## 2 Methods

### 2.1 Experimental approach

This intervention study uses a nonrandomized controlled trial with a pre–post design. Data collection was conducted during the artistic gymnasts’ preseason training periods (September–October and January–March), when training focuses on the development of physical fitness. Over a period of 6 weeks, the intervention group completed a HIIT, which was integrated into the normal gymnastics training, while the control group performed normal gymnastics training including their previously used programs for physical fitness development. The training duration (min per week) of the two groups was kept the same, as the time needed for HIIT was reduced from the normal gymnastics training of the HIIT group. One week before and two weeks after the intervention, the pre-test and post-test were scheduled.

### 2.2 Subjects

Twenty-five male artistic gymnasts (age: 15.0 ± 1.5 years; weight: 51.2 ± 10.6 kg; height 160.0 ± 10.4 cm) were recruited for this study. Due to different training locations, the athletes were not randomly assigned to a group; instead, the groups were divided according to the respective training location. The intervention group included 14 gymnasts (age: 14.4 ± 1.3 years; weight: 47.9 ± 9.2 kg; height: 157.4 ± 10.2 cm) and the control group 11 gymnasts (age: 15.8 ± 1.4 years; weight: 55.5 ± 11.2 kg; height: 163.3 ± 10.2 cm). The inclusion criteria for the athletes were: 1) healthy male artistic gymnasts, 2) age between 13 and 18 years, 3) membership of a regional or national squad, and 4) start of competitive gymnastics training at ≤6 years of age. The weekly training time of the athletes was ≥20.0 h. Prior to the study, the coaches and athletes were informed about the procedure and possible risks of the study. All athletes or their parents signed an informed consent before the start of the study. The experimental protocol was approved by the Ethics Committee of the Faculty of Cultural, Social and Educational Sciences at Humboldt-Universität of Berlin (HU-KSBF-EK_2022_0021) and was conducted in accordance with the Declaration of Helsinki.

### 2.3 Tests and measurements

#### 2.3.1 Pre- and post-tests

The pre- and post-tests consisted of two gymnastics-specific endurance tests and a gymnastics routine each on floor and pommel horse. These tests were performed on two consecutive days, with two tests per day. A 90-s repeated jump test (RJT) and a maximum circle test (CT) were used for the floor-specific and pommel horse-specific endurance performance, respectively (Figure 1).

The CT is a classical endurance test in gymnastics that determines the maximum number of circles (*C*_max_). Depending on the gymnasts’ abilities, the CT was performed on pommel horse or competition mushroom. Gymnasts who performed the pre-test on the mushroom also performed the post-test on the mushroom. For the RJT, a 12 × 12 m competition floor (Moscow, Spieth Gymnastics, Altbach, Germany) was used. The jump height was continuously recorded during the RJT using a girder bridge construction (29) with a light barrier system (Optojump Next, Microgate Srl, Bolzano, Italy). Taking into account the height of the light barrier system above the floor, the exported ground and flight times were corrected accordingly. To make the RJT as specific to gymnastics as possible, the artistic gymnasts were instructed to jump as high as possible on each jump, but with a short contact time and free use of the arms (30). The floor routine was performed on a 12 × 12 m competition floor (Moscow, Spieth Gymnastics, Altbach, Germany) and consisted of 10 acrobatic lines and one non-acrobatic element. In accordance with the characteristics of the sport, each gymnast performed individual acrobatic lines and non-acrobatic elements, which were identical in the pre- and post-test. The pommel horse routine consisted of 4 stations where different elements were performed. The stations involved were: a) 2 times front and back crossover travel with circles on the pommel horse without pommels, b) 20–30 times circles on the pommel horse or mushroom, c) 10 times scissors left and right, and 4) 20–30 times circles on the pommel horse or mushroom. Depending on the performance level, a different number of circles were completed at stations b and d, which, however, were identical in the pre- and post-test. Between the individual stations, the gymnasts left the apparatus briefly to move on to the next station. The execution of the floor and pommel horse routines were not evaluated, but the gymnasts were instructed to perform their routine in good quality. All four tests (including the pre- and post-load phases) were video-recorded (GC-PX100, JVCKENWOOD GmbH, Bad Vilbel, Germany) to determine the exact start and end as well as the difficulty score for the floor routine and the number of circles.

#### 2.3.2 Measurement of physiological data

During the RJT and CT, as well as the floor and pommel horse routines, breath-by-breath oxygen consumption (VO_2_) and heart rate (HR) were recorded using a portable telemetric spiroergometry system (VO2 Master, VO2 Master Health Sensors Inc, Vernon, Canada) coupled with an HR sensor (H9, Polar Electro Oy, Kempele, Finland). Prior to all tests, the spiroergometry system was calibrated with room air and a defined air volume of 3 L in accordance with the manufacturer's instructions. In the run-up to the study, the VO2 Master spiroergometry system was proofed to be valid (MPE 6.7% for VO_2_ and 7.6% for VE), reliable (SEM between 3.8 and 7.7% for VO_2_ parameters), and practically suitable in gymnastic-specific short-term exercise (e.g., circle test) (31). Before the start of the tests, the gymnasts had sufficient time to practice and become accustomed wearing the spiroergometry equipment. After the familiarization phase, a short rest phase was implemented to restore the resting level of the physiological parameters. Between the rest period and the start of the test, there was a 1-min pre-start phase, during which HR, VO_2_, and pre-start lactate were taken, and the video recording was started. VO_2_ and HR were measured during the 1-min pre-start phase, the test and the 8-min post-load phase. Due to the conditions in the gyms, the gymnasts had to walk a short distance after the test (walking phase) to sit on a chair. This walking phase lasted approximately 10 s and was considered in the calculation of the energy supply due to the different physiological conditions (32). To determine the blood lactate concentration (BLC), 20 μl of capillary blood was taken from the hyperemic earlobe using an end-to-end glass capillary in the pre-start phase, immediately after the end of the test and in the 1st, 3rd, 5th and 7th min of post-exercise. The capillary blood was then analyzed with a blood analyzer (SuperGl, Dr. Müller Gerätebau, Freital, Germany) to determine the BLC. 30 s after the end of the tests, the subjects had to report their subjective perception of exertion (RPE) using the 6–20 Borg scale (33).

### 2.4 Data analysis

To improve the underlying characteristics of the VO_2_ and HR data, occasional erroneous breaths caused by swallowing, coughing, sighing, etc. were manually eliminated, and the VO_2_ and HR data were then interpolated to 1-second values using the cubic spline method (OriginPro 8.0, Origin Laboratory Corp). The peak HR and peak VO_2_ of the floor and pommel horse routines were defined as the mean value of the last 5 s of the routines, the mean HR and mean VO_2_ as the mean value over the entire routine. The maximum number of circles in the CT were determined by manual counting. The parameters of the RJT were defined as follows:

- Peak jump height (Jump_peak_) = Highest value of the jump height over the entire 90 s
- Mean jump height (Jump_mean_) = Average jump height over the entire 90 s
- Jump height between 0 and 10 s (Jump_10_) = Average jump height over the first 10 s
- Jump height between 80 and 90 s (Jump_90_) = Average jump height over the last 10 s

The determination of the O_2_ deficit in the RJT and CT is based on Bearden and Moffatt (34) by monoexponentially curve fitting of the filtered VO_2_ data using non-linear least squares (OriginPro 8.0, Origin Laboratory Corp) (Equation 1):

(1) $$VO_{2}(t)=VO_{2\mathrm{rest}}+A_{1}\times (1-e^{-\left(t-TD\right)/\tau_{1}})$$

where VO_2_ (*t*) is the VO_2_ at any time (*t*), VO_2rest_ is the VO_2_ resting value before exercise, *A*_1_ is the increase in VO_2_ above the resting value, *τ*_1_ is the time constant (defined as the time required for VO_2_ to increase to a value equal to 63% of *A*_1_), and TD is the time delay. Curve fitting was conducted from the start of exercise to the end of exercise, with the first 20 s of VO_2_ data after the start of exercise (i.e., the phase I response) being removed (22). Since a different load time could occur between the pre-test and post-test in the CT due to an increase/reduction of the maximum number of circles the data in both tests was adjusted to the shorter load time. VO_2rest_ was calculated with the Benedict-Harris formula (35). Using the curve model, the O_2_ deficit was then calculated as (Equation 2) (34):

(2) $$O_{2\mathrm{DEF}\;}=\mathrm{load}\;\mathrm{time}\times (VO_{2\mathrm{rest}}+A_{1})-\int_{\mathrm{Start}}^{\mathrm{End}}\mathrm{equation}1\;df$$

Energy supply for the floor and pommel horse routines was calculated using the PCr-LA-O_2_ method (36). *W*_AER_ was calculated from the VO_2_ over the resting metabolic rate and the caloric equivalent as follows (Equation 3):

(3) $$\begin{array}{r} W_{\mathrm{AER}}\left[\mathrm{kJ}\right]=\dot{V}O_{2}(\mathrm{ml})\times \mathrm{caloric}\;\mathrm{equivalent}(J\times ml^{-1})\times 1,000^{-1} \end{array}$$

VO_2_ above resting metabolic rate was determined as the area under the curve of actual VO_2_ minus resting metabolic rate. Resting metabolic rate (VO_2rest_) was calculated using the Benedict-Harris formula (35) to exclude the influence of sympathetic arousal. The required caloric equivalent was defined as 20.9 J·ml^−1^ (37). *W*_BLC_ was calculated from the highest change in BLC (ΔBLC), the oxygen-lactate equivalent of 3.0 ml-O_2_·kg^−1^·mmol^−1^·L^−1^ and body weight (Equation 4):

(4) $$\begin{array}{rl} W_{\mathrm{BLC}}\left[\mathrm{kJ}\right] & =\Delta \mathrm{BLC}\times 3.0(\mathrm{ml}\times O_{2}\;\times \;kg^{-1}\;\times L^{-1}\;)\\ & \;\times caloric\;\mathrm{equivalent}(J\times ml^{-1})\times 1,000^{-1} \end{array}$$

*W*_PCr_ was calculated based on the fast component of post-exercise oxygen uptake (VO_2PCr_) and determined using a biexponential curve fit of excessive post exercise oxygen consumption (EPOC) (Equation 5):

(5) $$VO_{2\mathrm{EPOC}}\left[t\right]=A_{1}\times e^{-(t-TD)/\tau_{1}}+A_{2}\times e^{-(t-TD)/\tau_{2}}+VO_{2\mathrm{rest}}$$

where VO_2_ (*t*) is the VO_2_ at time t, VO_2rest_ is the VO_2_ of the resting metabolism, A_1_ and A_2_ are the amplitude of the fast and slow components, *τ*_1_ and *τ*_2_ are the associated time constants, and TD is a time delay. To account for the above-mentioned walking phase (end of exercise—sitting chair), VO_2PCr_ (first term of Equation 5) was divided into 2 phases. This was done because the resynthesis of creatine phosphate (PCr) can be reduced up to 20% during the walking phase (32). The split time was defined as the end of the walking phase (*t*1) and determined manually from the video recordings. The VO_2_ of phase 1 (*P*1) was calculated using the integral reduced by 20% and the VO_2_ of phase 2 (*P*2) was then determined using the integral from *t*1 to the end of the fast component (*t*2) (Equations 6, 7):

(6) $$VO_{2P1}\;(t)=0.8\times \;\int_{0}^{t1}A_{1a}\times e^{-(t-TD)/\tau_{1}}$$

(7) $$VO_{2P2}\;(t)=\;\int_{t1}^{t2}A_{1a}\times e^{-(t-TD)/\tau_{1}}$$

Finally, W_PCr_ was calculated from the sum of phase 1 and phase 2 multiplied by the caloric equivalent (Equation 8):

(8) $$W_{\mathrm{PCr}}(\mathrm{kJ})=(VO_{2P1}(\mathrm{ml})+VO_{2P2}(\mathrm{ml}))\times \mathrm{caloric}\;\mathrm{equivalent}\;(J\times ml^{-1})\times 1,000^{-1}$$

The total energy (*W*_TOTAL_) was finally calculated as the sum of the contributions of the individual energy systems (Equation 9) and the anaerobic energy contribution (*W*_ANAER_) as the sum of *W*_BLC_ and *W*_PCr_ (Equation 10):

(9) $$W_{\mathrm{TOTAL}}[\mathrm{kJ}]=W_{\mathrm{AER}}+W_{\mathrm{BLC}}+W_{\mathrm{PCr}}$$

(10) $$W_{\mathrm{ANAER}}[\mathrm{kJ}]=W_{\mathrm{BLC}}+W_{\mathrm{PCr}}$$

All energy shares were calculated in kJ and are presented in relative (% of *W*_TOTAL_) numbers

### 2.5 High intensity interval training

Two HIIT sessions took place per week with a time interval of at least two days between the sessions. The HIIT consisted of two sets with a 5-min passive recovery between the sets. For each set, eight exercise intervals with an exercise duration of 60 s and a passive recovery of 60 s were performed. The exercise intensity was set at 90% of the gymnasts’ individual maximum heart rate (HR_max_), which was determined using the Fox formula (38). The heart rate sensor (H9, Polar Electro Oy, Kempele, Finland) and the “Polar Team” application on a tablet (iPad 6, Apple, Cupertino, USA), which displays the heart rate data in real-time, were used to monitor the exercise intensity during HIIT. Four upper and four lower body exercises were selected for the HIIT, which were performed two times in each set (Table 1). The duration of each exercise was 30 s, allowing two exercises (one exercise set) to be performed in one exercise interval. The exercises were selected based on the following criteria: a) high equivalence to the load profile of the pommel horse and floor b) achievement of the corresponding load intensity.

**Table 1:** Interval structure and exercise description of the high intensity interval training.

	Interval structure per set	Exercise block	Exercise description	body section
Two sets with 5-min passive recovery	8 × 60 (30 + 30) seconds work per exercise block+60 s rest between the exercise blocks	1.	12-meter push gymnastic block (2,0 m × 1,0 m × 1,0 m; 52 kg)	Lower body
			Alternating foot jumps on a 40 cm gymnastic box	
		2.	Two-legged obstacle jumping on the floor	
			12-m sprints	
		3.	Battle Rope with alternate beating of the arms	Upper body
			Push-ups	
		4	Kettlebell Swing	
			Back push-up with hands on a 40 cm gymnastic box	

### 2.6 Statistical analysis

Descriptive statistics were performed and the data are presented as median [Interquartile range (IQR)]. The gain score method was used to test for significant differences between the groups. The gain is the difference between the post- and the pre-test results (39). Normal distribution and homogeneity of variance of the gains were assessed using the Shapiro–Wilk test and the Levene test and could not be confirmed. Therefore, the data were then analyzed for differences in the central tendencies using one-sided Mann–Whitney *U* tests. The one-sided Mann–Whitney *U* test was conducted on the basis of the directional hypotheses that the intervention group achieved a greater improvement in the relevant parameters compared to the control group. Before interpreting the results of the Mann–Whitney *U* test, the distribution in the groups was analyzed for all parameters using the Kolmogorov–Smirnov test and no different distribution was found. The effect size (Pearson *r*) was used to categorize the significance of the results, evaluated as trivial (0–0.09), small (0.10–0.29), medium (0.30–0.49) and large (≥ 0.50) (40). In addition, the 95% confidence interval of Pearson r was calculated. Spearman's Rho was used to analyze the statistical correlations between the gains of the parameters. Statistical analyses were performed using Excel 2016 (Microsoft, Redmond, USA), Jamovi [version 2.3]) and RStudio [version 4.4.0]. The level of statistical significance was set at 5% (*p* < 0.05) for all analyses.

## 3 Results

Tables 2, 3 show the results of the RJT and CT. The integrated HIIT in the normal gymnastics training led to a significantly greater improvement in Jump_mean_ (*Z* = 17.00; *p* = 0.011; *r* = −0.511) and Jump_90_ (*Z* = 13.00; *p* = 0.005; *r* = −0.587) in the RJT. In addition, the intervention group showed a significantly greater reduction in the O_2_ deficit (*Z* = 10.00; *p* = 0.002; *r* = −0.644), *τ*_1_ (*Z* = 11.00; *p* = 0.002; *r* = −0.625), and delta lactate (*Z* = 23.00; *p* = 0.045; *r* = −0.399) in the RJT (Table 2). In the CT no significant difference was found between the groups for these parameters (Table 3). There was also no significantly greater improvement in *C*_max_ (*Z* = 48.00; *p* = 0.575; *r* = 0.197) with the integrated HIIT compared to normal gymnastics training (Table 3).

**Table 2:** Statistical parameters in the performance and physiological parameters of the intervention (HIIT) and control group (CON) in the repeated jump test.

Parameters	HIIT			CON			Significance	Effect size	CI 95%
	Pre	Post	difference	Pre	Post	difference			
	Median [IQR]			Median [IQR]			*p*	Pearson r	Lower upper
Jump_peak_ [cm]	45.0	47.3	2.58	47.0	50.3	0.335	0.300	0.133 Small	−0.369
	[12.6]	[8.12]	[5.58]	[6.94]	[6.47]	[1.16]			0.571
Jump_mean_ [cm]	33.4	39.2	2.51	39.7	38.9	−0.20	0.013*	0.511	0.098
	[8.91]	[6.21]	[3.43]	[4.90]	[5.31]	[0.65]		Large	0.830
Jump_10_ [cm]	41.5	44.3	1.46	44.7	45.6	0.04	0.329	0.114	−0.344
	[11.4]	[7.51]	[5.23]	[5.96]	[5.24]	[2.63]		Small	0.551
Jump_90_ [cm]	31.0	35.1	1.37	35.8	36.0	−0.89	0.005*	0.587	0.188
	[7.97]	[5.03]	[5.29]	[6.08]	[7.42]	[0.95]		Large	0.847
Δ BLC [mmol/L]	5.98	5.29	−0.64	4.81	5.44	0.625	0.045*	−0.399	−0.762
	[2.59]	[2.75]	[1.38]	[1.10]	[1.17]	[1.17]		Medium	0.043
RPE	15.0	16.0	1.0	16.0	16.05	1.0	0.273	−0.155	−0.569
	[1.0]	[1.5]	[1.0]	[2.0]	[1.5]	[1.0]		Small	0.317
O_2_ deficit [ml/kg]	18.3	16.6	−1.21	19.0	19.3	0.75	0.002*	−0.544	−0.803
	[4.29]	[3.34]	[1.56]	[5.45]	[4.69]	[1.76]		Large	−0.139
A_1_ [ml/min/kg]	44.3	43.6	−0.07	47.7	47.5	0.54	0.054	−0.379	−0.723
	[9.75]	[10.2]	[2.09]	[8.58]	[7.61]	[2.34]		Medium	0.078
*τ*_1_ [s]	31.9	29.8	−3.83	32.4	32.6	1.33	0.002*	−0.625	−0.837
	[9.24]	[7.62]	[4.64]	[12.6]	[12.1]	[3.47]		Large	−0.301

SD, standard deviation; Jump_peak_, Highest value of the jump height over the entire 90 s in the repeated jump test; Jump_mean_, Average value of the jump height over the entire 90 s in the repeated jump test; Jump_10_, Average value of the jump height over the first 10 s in the repeated jump test; Jump_90_, Average value of the jump height over the last 10 s in the repeated jump test; BLC, blood lactate concentration; RPE, rate of perceived exertion; A_1_, amplitude of VO_2_ kinetic; τ_1_, time constant of VO_2_ kinetics.

* Significant difference in the gain between the intervention and control group.

**Table 3:** Statistical parameters in the performance and physiological parameters of the intervention (HIIT) and control group (CON) in the maximum circle test.

Parameters	HIIT			CON			Significance	Effect size	CI 95%
	Pre	Post	difference	Pre	Post	difference			
	Median [IQR]			Median [IQR]			*p*	Pearson r	lower upper
*C*_max_ [numbers]	55.5	55.7	4.5	58.5	63.5	5.5	0.575	−0.034	−0.483
	[17.0]	[19.5]	[7.25]	[21.0]	[21.0]	[7.0]		Trivial	0.425
Δ BLC [mmol/L]	6.71	7.33	0.03	7.05	7.55	0.61	0.163	−0.288	−0.633
	[2.04]	[2.91]	[1.79]	[0.69]	[1.07]	[0.85]		Small	0.248
RPE	15.50	15.00	0.50	16.00	16.50	0.50	0.439	−0.043	−0.476
	[2.50]	[1.00]	[1.75]	[1.50]	[1.75]	[1.75]		Trivial	0.400
O_2_ deficit [ml/kg]	8.50	9.04	−0.36	8.43	7.98	−0.05	0.633	0.068	−0.418
	[2.49]	[1.83]	[0.75]	[3.10]	[3.02]	[0.47]		Trivial	0.487
A_1_ [ml/min/kg]	32.0	32.4	−0.34	31.6	29.6	0.0	0.575	0.034	−0.443
	[5.23]	[4.36]	[2.64]	[9.76]	[9.10]	[1.53]		Trivial	0.487
τ_1_ [s]	29.3	29.1	−1.73	23.3	19.7	0.00	0.395	−0.068	−0.530
	[9.17]	[7.88]	[3.30]	[11.0]	[7.72]	[3.85]		Trivial	0.384

SD, standard deviation; *C*_max_, maximum numbers of circles in the maximum circle test; BLC, blood lactate concentration; RPE, rate of perceived exertion; A_1_, amplitude of VO_2_ kinetic; τ_1_, time constant of VO_2_ kinetics.

* Significant difference in the gain between the intervention and control group.

Tables 4, 5 present the physiological and energetic parameters of the floor and pommel horse routines. On the floor, the intervention group showed a significantly greater increase in VO_2mean_ (*Z* = 18.00; *p* = 0.027; *r* = −0.461) and VO_2peak_ (*Z* = 16.00; *p* = 0.017; *r* = −0.503) (Table 4.). On pommel horse, a significant reduction in HR_mean_ (*Z* = 10.00; *p* = 0.020; *r* = −0.551) and HR_peak_ (*Z* = 9.00; *p* = 0.009; *r* = −0.604) were achieved by the intervention group (Table 5). Regarding energy supply, the HIIT leads to a significantly greater increase in aerobic metabolism (*Z* = 14.00; *p* = 0.010; *r* = −0.544) and a significantly greater reduction (*Z* = 14.00; *p* = 0.010; *r* = −0.544) in anaerobic metabolism during floor routine. In addition, a significantly greater increase in aerobic metabolism (t = 9.00; *p* = 0.007; *r* = −0.604) and a significantly greater reduction was detected for anaerobic metabolism on the pommel horse (*Z* = 9.00; *p* = 0.007; *r* = −0.604). The HIIT group was able to increase the aerobic energy supply on floor by 2.69 ± 4.13% on average and at the same time reduce the anaerobic energy supply by −2.68 ± 4.13%. On the pommel horse, the aerobic energy supply in the HIIT group increased by 2.78 ± 2.16% on average with a simultaneous reduction in the anaerobic energy supply of −2.77 ± 2.16%. For both floor (*Z* = 15.00; *p* = 0.013; *r* = −0.461) and pommel horse (*Z* = 13.00; *p* = 0.025; *r* = −0.499), the intervention group shows a significant reduction in WPCr.

**Table 4:** Statistical parameters of the physiological and energetic parameters of the intervention (HIIT) and control group (CON) on floor.

Parameters	HIIT			CON			Significance	Effect size	CI 95%
	Pre	Post	difference	Pre	Post	difference			
	Median [IQR]			Median [IQR]			*p*	Pearson r	lower upper
mean HR [bpm]	168	168	−0.75	172	176	1.03	0.204	−0.210	−0.634
	[10.3]	[4.50]	[6.20]	[13.4]	[22.4]	[6.40]		Small	0.295
peak HR [bpm]	182	179	−2.25	184	180	0.05	0.252	−0.187	−0.616
	[5.08]	[6.20]	[5.44]	[13.4]	[15.9]	[5.04]		Small	0.330
mean VO_2_ [ml/kg]	38.7	40.0	1.38	45.4	45.6	0.52	0.027*	0.460	NA
	[6.53]	[7.10]	[2.20]	[1.58]	[1.81]	[1.49]		Medium	NA
peak VO_2_ [ml/kg]	50.0	51.3	1.51	55.8	55.1	−0.92	0.017*	0.502	0.059
	[6.52]	[8.31]	[1.83]	[3.62]	[1.58]	[2.07]		Large	0.799
Δ BLC [mmol/L]	8.02	7.69	0.04	6.41	6.23	−0.11	0.483	0.021	−0.467
	[1.06]	[1.93]	[1.32]	[0.97]	[1.62]	[0.60]		Trivial	0.445
RPE	16.0	15.5	−1.0	17.0	16.0	0.0	0.132	−0.273	−0.772
	[1.75]	[1.75]	[1.50]	[1.50]	[1.25]	[0.50]		Small	0.173
*W*_AER_ [%]	47.4	49.3	1.91	56.0	53.4	−1.80	0.010*	0.544	0.147
	[4.64]	[5.75]	[5.37]	[10.8]	[10.4]	[2.55]		Large	0.804
*W*_ANAER_ [%]	52.6	49.8	−1.91	44.0	46.6	1.79	0.010*	−0.544	−0.801
	[4.63]	[7.36]	[5.37]	[10.8]	[10.4]	[2.55]		Large	−0.161
*W*_BLC_ [%]	20.6	19.7	0.01	17.4	15.5	0.00	0.552	0.021	−0.445
	[5.37]	[5.54]	[5.74]	[2.55]	[4.16]	[5.41]		Small	0.533
*W*_PCr_ [%]	33.4	28.8	−1.84	27.1	28.9	1.02	0.013*	−0.460	−0.761
	[7.91]	[7.56]	[6.53]	[11.0]	[8.80]	[2.93]		Medium	0

SD, standard deviation; HR, heart rate; VO_2_, oxygen consumption; BLC, blood lactate concentration; RPE, rate of perceived exertion; *W*_AER_, relative energy contribution from the aerobic metabolism; *W*_ANAER_, relative energy contribution from the anaerobic metabolism; *W*_BLC_, relative energy contribution from anaerobic-lactic metabolism; *W*_PCr_, relative energy contribution from anaerobic-alactic metabolism; NA, not predictable.

* Significant difference in the gain between the intervention and control group.

**Table 5:** Statistical parameters of the physiological and energetic parameters of the intervention (HIIT) and control group (CON) on pommel horse.

Parameters	HIIT			CON			Significance	Effect size	CI 95%
	Pre	Post	difference	Pre	Post	difference			
	Median [IQR]			Median [IQR]			*p*	Pearson r	lower upper
mean HR [bpm]	172	167	−3.41	172	172	−0.35	0.020*	−0.537	−0,842
	[15.6]	[11.5]	[3.82]	[17.6]	[18.3]	[2.51]		Large	−0,032
peak HR [bpm]	182	176	−4.65	188	187	0.50	0.009*	−0.605	−0.842
	[7.68]	[6.35]	[3.95]	[13.9]	[11.9]	[4.50]		Large	−0.214
mean VO_2_ [ml/kg]	35.9	37.2	1.28	35.7	34.1	0.45	0.080	0.368	NA
	[9.81]	[9.00]	[1.20]	[5.27]	[4.99]	[1.64]		Medium	NA
peak VO_2_ [ml/kg]	43.2	46.3	2.60	39.3	41.2	0.82	0.191	0.236	−0.290
	5.87	8.89	2.00	6.57	10.1	2.76		Small	0.678
Δ BLC [mmol/L]	6.30	4.98	−0.42	7.21	6.60	−0.53	0.439	0.053	−0.445
	[1.25]	[2.46]	[1.07]	[0.87]	[0.50]	[1.18]		Trivial	0.550
RPE	15.0	15.0	−1.0	17.0	17.0	0.0	0.141	0.282	−0.690
	[2.0]	[2.0]	[1.5]	[1.5]	[1.25]	[2.50]		Small	0.206
*W*_AER_ [%]	56.3	57.0	2.55	57.2	56.2	−1.17	0.007*	0.605	0.212
	[7.87]	[9.39]	[2.33]	[5.73]	[4.49]	[5.67]		Large	0.838
*W*_ANAER_ [%]	43.8	43.0	−2.55	42.8	43.8	1.17	0.007*	0.605	−0.840
	[7.87]	[9.40]	[2.32]	[5.73]	[4.48]	[5.68]		Large	−0.214
*W*_BLC_ [%]	17.1	15.0	−1.27	17.3	17.2	−1.27	0.0561	0.026	−0.495
	[7.37]	[4.05]	[3.07]	[3.07]	[4.54]	2.03]		Trivial	0.555
*W*_PCr_ [%]	27.0	27.7	−0.45	24.4	26.7	0.81	0.025*	−0.500	0.838
	[3.03]	[8.52]	[3.93]	[7.12]	[10.6]	[4.86]		Large	−0.014

SD, standard deviation; HR, heart rate; VO_2_, oxygen consumption; BLC, blood lactate concentration; RPE, rate of perceived exertion; *W*_AER_, relative energy contribution from the aerobic metabolism; *W*_ANAER_, relative energy contribution from the anaerobic metabolism; *W*_BLC_, relative energy contribution from anaerobic-lactic metabolism; *W*_PCr_, relative energy contribution from anaerobic-alactic metabolism; NA, not predictable.

* Significant difference in the gain between the intervention and control group.

**Figure 2:**
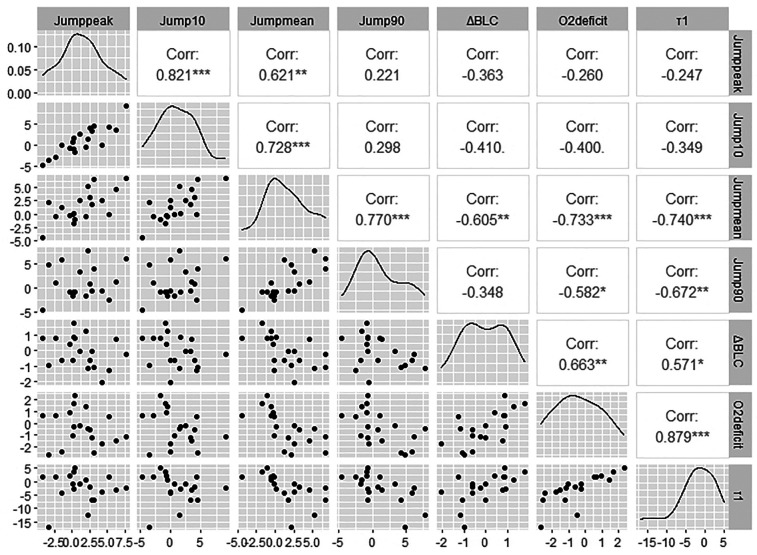
Correlogram for the comparison of the performance and physiological parameters in the repeated jump test.

Looking at the physiological factors for the effectiveness of HIIT, the correlation analyses revealed a significant negative correlation (Rho = −0.73; *p* =  < 0.001) between the difference in O_2_ deficit and the difference in Jump_mean_ in the RJT (Figure 2). There is also a significant negative correlation for the difference in O_2_ deficit and the difference in Jump_90_ (Rho = −0.58; *p* = 0.010). The difference in *τ*_1_ also shows a significant negative correlation with the difference in Jump_mean_ in the RJT (Rho = −0.74; *p* = < 0.001) as well as with the difference in Jump_90_ (Rho = −0.67; *p* = 0.002).

**Figure 3:**
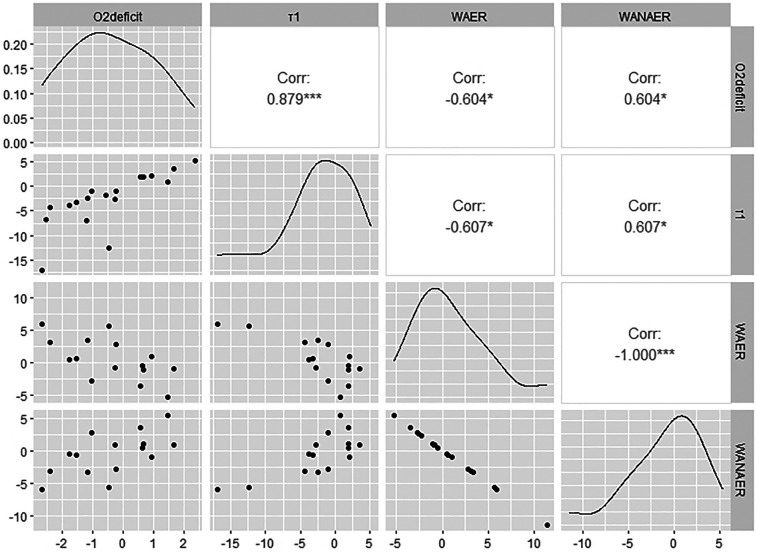
Correlogram for the comparison of the physiological parameters in the repeated jump test and the energy parameter on floor.

Furthermore, there is a significant negative correlation (Rho = −0.60; *p* = 0.020) between the difference in O_2_ deficit in the RJT and the difference in the relative *W*_AER_ on floor (Figure 3). There is also a significant negative correlation (Rho = −0.61; *p* = 0.019) for the difference in *τ*_1_ in the RJT and the difference in the relative *W*_AER_ on floor (Figure 3).

**Figure 4:**
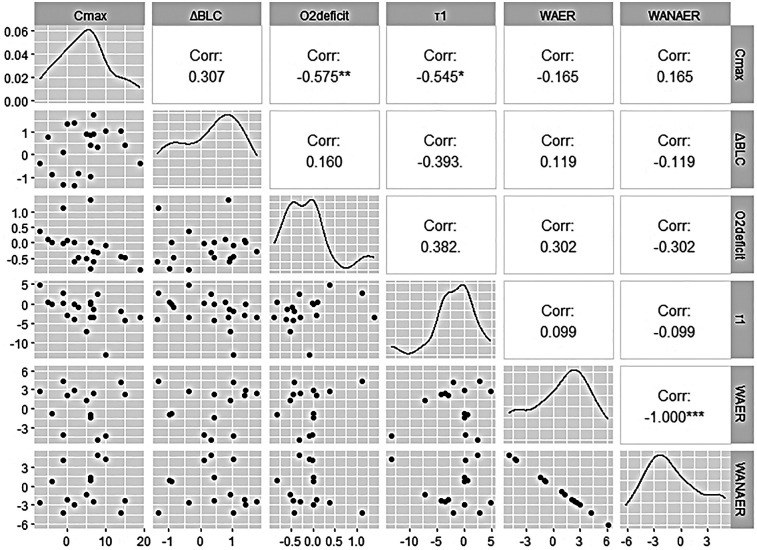
Correlogram for the comparison of the physiological parameters in the circle test and the energy parameter on pommel horse.

In CT, there is a significant negative correlation for the difference in O_2_ deficit and the difference in *C*_max_ (Rho = −0.58; *p* = 0.008) and a significant negative correlation for the difference *τ*_1_ and the difference in *C*_max_ (Rho = −0.55; *p* = 0.013) (Figure 4). No significant correlations were found for the differences in the physiological parameters in the CT and the differences in the relative energy fractions on pommel horse.

## 4 Discussion

The study aimed to examine the effectiveness of HIIT integrated into normal gymnastics training on gymnastics-specific endurance performance and energy supply during floor and pommel horse routines. Contrary to the first hypothesis (H1), the HIIT only leads to a greater improvement in gymnastics-specific endurance performance in the RJT (i.e., Jump_mean_), however, the HIIT did not increase the maximum number of circles in the CT. The increase in Jump_mean_ is in contrast to a previous study that reported no effects of HIIT on mean jump height in a 30-second RJT (seven young female sub-elite aerobic gymnasts) compared to a control group performing normal acrobatic training (41). The reason for the inconsistent results of the studies could be explained by the different loading times of the RJT and thus the different energy supply. The anaerobic part of the energy supply is presumably higher during a 30 s RJT compared to a 90 s RJT, so the effects of HIIT (i.e., increase in aerobic power) on the average jump performance did not fully apply. Improving the mean jump height in an RJT is important as gymnasts must be able to generate a similar amount of net momentum and force throughout the floor routine (30). The aforementioned positive association is strengthened by the study of Marina and Rodríguez (30), where the mean jump height in a 60 s RJT is positively correlated (Rho = 0.98) with the judge's score on the floor in twenty teenage elite artistic gymnasts. A greater improvement in peak jumping performance, expressed in Jump_peak_ could not be achieved with the integrated HIIT. However, both groups achieved an improvement in Jump_peak_ in the post-test (HIIT = +1.62 ± 3.92 cm; CON = +1.15 ± 1.83 cm), which can presumably be attributed to the normal gymnastics training in the preparation phase. As the aim of the study was to improve the gymnastics-specific endurance performance and thus Jump_mean_, this result is understandable. In a study with aerobic gymnasts, integrating HIIT into normal training also did not result in a significantly greater improvement in peak jumping power performance in a counter-movement jump compared to a control group with only normal acrobatic training (42). However, the same study showed a significantly greater improvement in peak jump power performance for the jump interval training (JIT) group compared to the control group with only normal training (42). So, if the training goal is to achieve a greater improvement in peak jumping performance compared to normal gymnastics or acrobatics training, JIT should be used. HIIT and JIT appear to produce different adaptations that should be used as specifically as possible, especially in high-performance sport. JIT requires explosive force generation, which more effectively stimulates fast-twitch muscle fibers than running intervals, resulting in an enhanced neuromuscular power, which is crucial for improving maximum jump height (42). HIIT, on the other hand, results in adaptations at physiological energetic levels (oxidative adaptations), which appears to be crucial for the increase in mean jumping power during RJT. The present study found a significant correlation between the increase in mean jump height and the reduction in the O_2_ deficit as well as between the increase in mean jump height and the reduction in the time constants of VO_2_ kinetics, which partially confirms the third hypothesis.

As mentioned previously, a greater increase in *C*_max_ through the integrated HIIT was not detected. Possible reasons for this could be related to the used CT or the designed HIIT. In addition to muscular fatigue, there may have been other influences on *C*_max_ in the CT. The fatigue at the end of the CT may have resulted in a poor grip and thus impaired balance, which could have resulted in the CT being stopped without complete fatigue being achieved. However, this effect can also be transferred to the control group. The analysis of the HR data from the HIIT shows that the required exercise intensity of 90% of HR_max_ was mostly not achieved in the upper body exercises. This can be explained by lower muscle mass in the upper body, which leads to differences in metabolic and cardiovascular responses compared to leg training (28). Arm cranking shows slower HR kinetics compared to leg training, probably due to lower sympathetic stimulation of the heart during arm training (43). Achieving a high intensity in HIIT is important as only exercise intensities near VO_2max_ allow for both large motor unit recruitment (i.e., type II muscle fibres) and attainment of near-to-maximal cardiac output, which, in turn, together represent signals for oxidative muscle fibre adaptation and myocardium enlargement (44). Therefore, it can be assumed that the training stimulus for an adaptation of aerobic performance in the upper body was not sufficient. Another explanation is that circles are too specific movements to be trained by the implemented HIIT. Although one study showed that HIIT can significantly increase maximum numbers of circles, the HIIT carried out in this study also included 40 s circles as one of the five exercises (20). In the present study, circles were not used as a HIIT exercise, as gymnasts are often affected by wrist problems due to pommel horse training. In addition to physiological and test-related factors, the non-randomized design and sample size may also have limited the statistical power to detect changes in *C*_max_.

The second research hypothesis, that semi-specific HIIT increases the relative aerobic energy contribution during floor and pommel horse routines, was confirmed. The greater increase in aerobic metabolism reduced anaerobic metabolism and thus offers the possibility of a) creating energetic performance reserves (conservation of PCr stores) and b) reducing factors of muscular fatigue. This provides the conditional basis for increasing exercise difficulty and ensures that a high exercise quality is maintained throughout the exercise. These aspects highlight the importance of developing specific endurance performance in artistic gymnastics. The modification of energy supply through HIIT is consistent with the results of other studies (45–47). Some of these studies showed that the change in energy supply also resulted in a reduced O_2_ deficit (22, 45, 46). In the present study, the O_2_ deficit was not calculated for the floor and pommel horse routines. However, there is a correlation between the reduction of the O_2_ deficit in the RJT and the modification of the metabolic components on the floor. This confirms the fourth hypothesis. The reduction in the O_2_ deficit can be attributed to a faster VO_2_ kinetic.

The energy data also show that the integrated HIIT primarily reduces the energy of the anaerobic alactic metabolism by increasing the aerobic metabolism. The anaerobic lactic metabolism showed no significant reduction in the relative energy content. Thus, the results are somewhat contrary to the results of Park and Yang (47), who reported that a 4-week integrated HIIT led to a significant reduction in the relative anaerobic lactic energy content in a high-intensity cardio yoga test (ten physically active individuals). This discrepancy may be due to the different energy demands of the different types of exercise. For example, the relative W_PCr_ for the floor and pommel horse routines examined in this study is around 30%, while for the high-intensity cardio yoga test it is around 10%. The fact that different types of exercise have different energy demands was also demonstrated by Kaufmann, Hoos (48), who showed that a 30-second RJT depends more on alactic and less on lactic energy contribution than a Wingate test. Because anaerobic alactic capacity is an important component of gymnastic-specific endurance (8, 9), reducing W_PCr_ is an important training adaptation. In this way, HIIT can be performed to save PCr, which can be used as an energy reserve for increasing exercise difficulty.

Based on the determined correlation, conclusions about the physiological reason for the effectiveness of HIIT in modifying energy supply and improving specific endurance performance on the floor can be made. Notably, an acceleration of VO_2_ kinetics is mainly due to an improvement in the intramuscular part (enzyme activity and content, and mitochondrial volume and content) of VO_2_. Aerobic power, expressed as maximum VO_2_, is made up of the power of the pulmonary, cardiovascular, and muscular systems (49). Therefore, it can be concluded that especially the intramuscular part of aerobic performance is an important component of gymnastic-specific endurance performance. Using this knowledge, future gymnastics-specific endurance training programs and performance tests can be designed more specifically to stimulate or identify corresponding physiological adaptations.

When interpreting the results, however, the following limitations of the study must be taken into account. The study was conducted with twenty-five male junior high-level gymnasts. Due to the small sample size, the generalizability of the results is limited. The selection of exclusively male junior high-level gymnasts limits the transferability to other groups of people (junior female gymnasts or senior male gymnasts), meaning that the validity and significance of the results can only be related to the group of people and performance level studied. Future studies with a larger participant group and an age- and gender-related expansion of the participant group are recommended to increase the transferability of the results. Instead of randomization, group selection in this study was based on the training location, which could not be realized otherwise in professional training practice. Nevertheless, the insufficient randomization leads to a reduction in internal validity and could have caused a selection or location effect. Further limitations of the study include the use of the Fox formula to determine HRmax, which is less accurate in adolescents, and the matching of the total training duration of the groups instead of the training volume. This approach was taken to enable the practicability of such a study in a professional sports context, as, for example, an additional HRmax test could not be performed due to time limitations. The experimental process was routine in a training competition, therefore, the available data should be compared with future studies. When calculating the energy contributions, it was assumed that the replenishment of PCr stores after exercise is entirely attributable to the aerobic system, whereby a possible minor contribution of the glycolytic system to PCr resynthesis was neglected (48). Another point that needs to be addressed is the calculation of the energy supply during the pommel horse routine. For the pommel horse routines, the VO_2_ data during the short breaks were neglected as a potential W_PCr_ contribution, as the recovery time was very short and the athletes jogged during the breaks, which maintained the increased VO_2_. Thus, there was no rest period in which the PCr could have been replenished, so these values were considered as oxidative phosphorylation energy (50). Since this study is mainly concerned with the difference between the intervention and control groups, the aspects of energy calculation can be neglected.

## 5 Conclusions

It was shown that HIIT resulted in a greater increase in the relative aerobic and a greater reduction in the relative anaerobic energy component during floor and pommel horse routines. The modification of the energy supply can have a positive influence, as it can create energetic performance reserves and reduce factors of muscular fatigue. It was also shown that HIIT increased Jump_mean_ in a 90-s RJT. The average jump height is an important performance parameter for gymnastic-specific endurance on the floor (30). HIIT fosters faster VO_2_ kinetics and thus a reduced O_2_ deficit. Based on this, it can be concluded that the muscular part of aerobic performance is an important component of gymnastics-specific endurance performance. Contrary to assumptions before the study, a greater improvement in *C*_max_ through HIIT could not be confirmed. This could be due to upper body exercises used in the HIIT, which mostly failed to achieve the desired exercise intensity. For this reason, future studies could examine whether a modified exercise selection or a modified load profile can positively influence endurance performance on the pommel horse.

## Data Availability

The raw data supporting the conclusions of this article will be made available by the authors, without undue reservation.
